# Synthetic data-driven overlapped neural spikes sorting: decomposing hidden spikes from overlapping spikes

**DOI:** 10.1186/s13041-024-01161-y

**Published:** 2024-11-28

**Authors:** Min-Ki Kim, Sung-Phil Kim, Jeong-Woo Sohn

**Affiliations:** 1 Translational Brain Research Center, Catholic Kwandong University, Gangneung, Republic of Korea; 2 Department of Biomedical Engineering, Ulsan National Institute of Science and Technology, Ulsan, Republic of Korea; 3 Department of Medical Science, Catholic Kwandong University, Gangneung, Republic of Korea

**Keywords:** Overlapping spikes, Synthetic data-driven approach, Spike sorting, Spike synchronization

## Abstract

Sorting spikes from extracellular recordings, obtained by sensing neuronal activity around an electrode tip, is essential for unravelling the complexities of neural coding and its implications across diverse neuroscientific disciplines. However, the presence of overlapping spikes, originating from neurons firing simultaneously or within a short delay, has been overlooked because of the difficulty in identifying individual neurons due to the lack of ground truth. In this study, we propose a method to identify overlapping spikes in extracellular recordings and to recover hidden spikes by decomposing them. We initially estimate spike waveform templates through a series of steps, including discriminative subspace learning and the isolation forest algorithm. By leveraging these estimated templates, we generate synthetic spikes and train a classifier using their feature components to identify overlapping spikes from observed spike data. The identified overlapping spikes are then decomposed into individual hidden spikes using a particle swarm optimization. Results from the testing of the proposed approach, using the simulation dataset we generated, demonstrated that employing synthetic spikes in the overlapping spike classifier accurately identifies overlapping spikes among the detected ones (the maximum F1 score of 0.88). Additionally, the approach can infer the synchronization between hidden spikes by decomposing the overlapped spikes and reallocating them into distinct clusters. This study advances spike sorting by accurately identifying overlapping spikes, providing a more precise tool for neural activity analysis.

**Supplementary Information:**

The online version contains supplementary material available at 10.1186/s13041-024-01161-y.

## Introduction

**Fig. 1:**
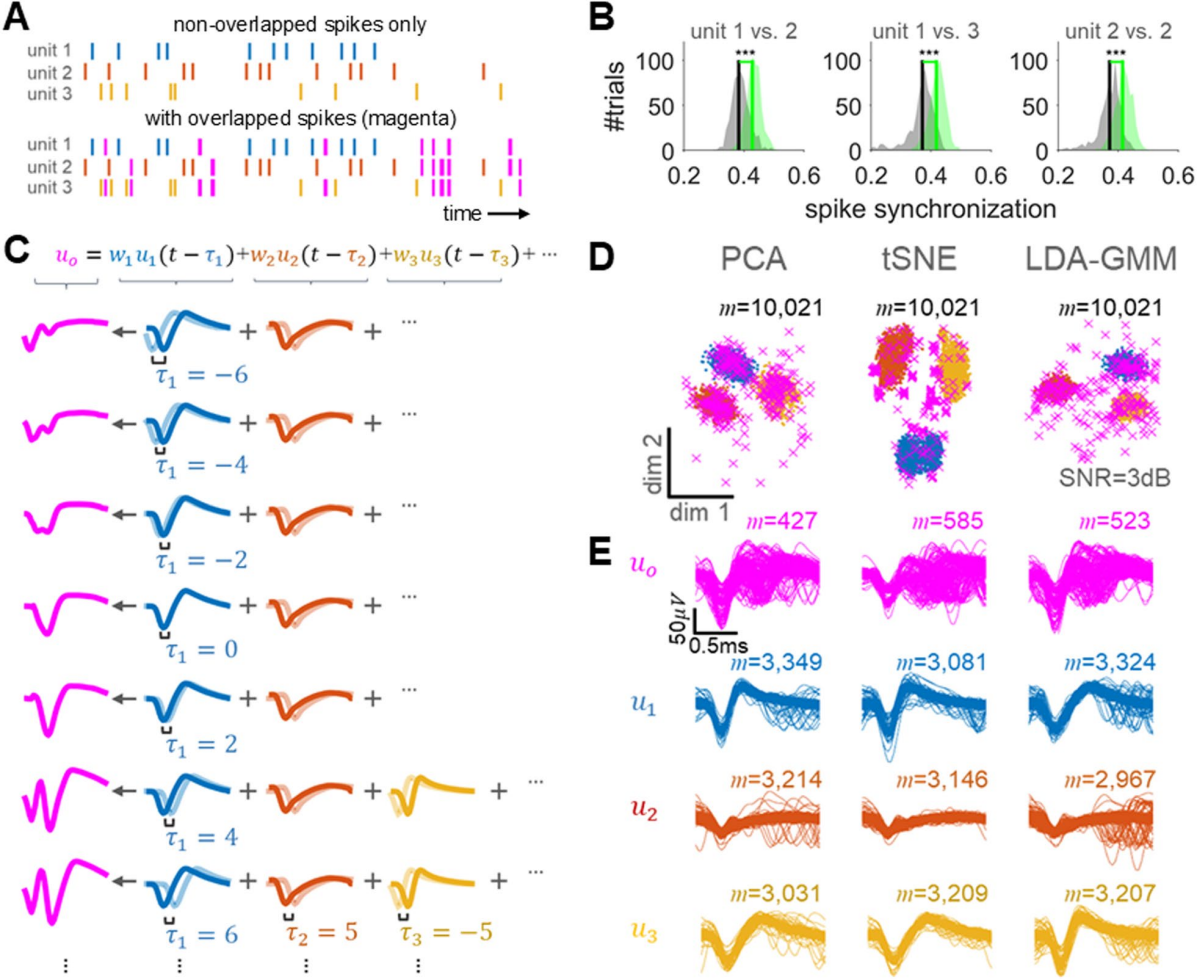
Examples of the impact of overlapping spikes on neural activity analysis. **A** Examples of spike trains with non-overlapping and overlapping waveforms. Solid blue-, orange-, and yellow-coloured lines are individual spike waveforms, and magenta coloured line denotes an overlapping spike. **B** Comparison of spike synchronization distributions between single units with non-overlapping (perfectly isolated) spikes and those with overlapping spikes. The dataset was generated through a synthetic spike generation procedure repeated 500 times (details provided in “Methods”). The shaded areas in grey and green represent the spike synchronization distributions for single units without and with overlapping spikes, respectively. Vertical solid lines indicate the median values of each distribution. Asterisks (***p < 0.001) denote statistically significant differences according to the Wilcoxon rank-sum test. **C** An overlapping spike model. **D** Visualization of feature distributions projected by three different subspace learning methods: PCA, tSNE, and LDA–GMM, and **E** examples of spike waveforms categorized based on each method

Electrophysiological research in neuroscience heavily relies on spike train analysis to decode neuronal activities and understand brain functions [1]. Precise spike sorting is crucial for understanding the firing patterns of neurons in response to endogenous and exogenous stimuli [2, 3]. However, a significant challenge is presented by overlapping spikes, where signals from multiple neurons are superimposed, complicating the identification of individual neuronal activities [4]. In particular, synchronization between neuronal spikes, which is considered an important aspect of temporal coding, serves as a crucial indicator of information processing [5–9]. However, paradoxically, in extracellular recordings, temporal synchronization of neurons concentrated around one electrode can result in overlapping spikes, posing challenges for the analysis of temporal coding [10, 11] (Fig. 1A, B). This issue becomes particularly prominent in scenarios involving dense electrode arrays or rapid firing sequences, highlighting the complexity of interpreting neuronal signals [10–14]. The widespread challenge of overlapping spikes has substantial implications for current research and the development of neuroengineering applications [15]. Traditional spike sorting methods, limited by their probabilistically analytical frameworks, often fall short in accurately isolating these superimposed neuronal signals [1, 4]. This limitation not only restricts our capacity to delve into the brain’s intricate dynamics but also impacts our ability to analyze the subtle mechanisms underlying neural processing [16, 17]. Therefore, there is an evident need for novel approaches that surpass the constraints of conventional techniques, facilitating more effective discrimination and the reuse of overlapping spikes.

The primary focus of this study is addressing the identification of overlapping spikes from detected signals. Overlapping spikes occur when the spike waveforms of individual neurons around a single electrode are detected simultaneously, influenced by factors such as waveform size (*w*), firing latency (*τ*), and distance from the electrode [18] (see Fig. 1C). Despite the potential similarity in firing latency, overlapping spikes are generally difficult to distinguish in the feature space commonly considered by the traditional spike sorting methods. For example, a principal component analysis (PCA) commonly serves as a feature extraction method for spike sorting, allowing the observation of spike clusters under relatively low-noise conditions [19, 20]. However, because it does not factor in noise, this method faces challenges with noise interference, hindering the accurate identification of overlapping spikes within a linear subspace [19, 20] (see Fig. 1D, E). On the other hand, a t-distributed stochastic neighbor embedding (tSNE) is a visualization technology using non-linear dimensionality reduction, which can identify spike clusters more clearly [21]. However, it does not provide clear clusters for identifying overlapping spikes. Recently, Keshtkaran and Yang proposed an approach that combines a linear discriminant analysis (LDA) and a Gaussian mixture model (GMM), optimizing the objective function of LDA through iterative feature extraction and clustering, which is called LDA–GMM [22]. LDA–GMM can estimate the subspace, which unambiguously transforms detected spikes into distinct features by effectively excluding outliers. Thus, it could be useful not only for estimating representative waveforms of a single unit, but also for detecting overlapping spikes (the excluded outliers) depending on the effect of GMM [22]. However, because a significant number of overlapping spikes could blend into the feature distribution, it may be difficult to clearly detect the characteristics of these overlapping spikes (Fig. 1D). In addition, a study explored deep learning techniques for estimating waveform feature spaces and classifying overlapping spikes, which are expected to offer high classification accuracy due to their robustness to noise [23]. While these techniques show promise, their primary validation on simulation data, treated as ground truth, calls into question the applicability of such methodologies in accurately capturing the complexities of real-world data.

**Fig. 2:**
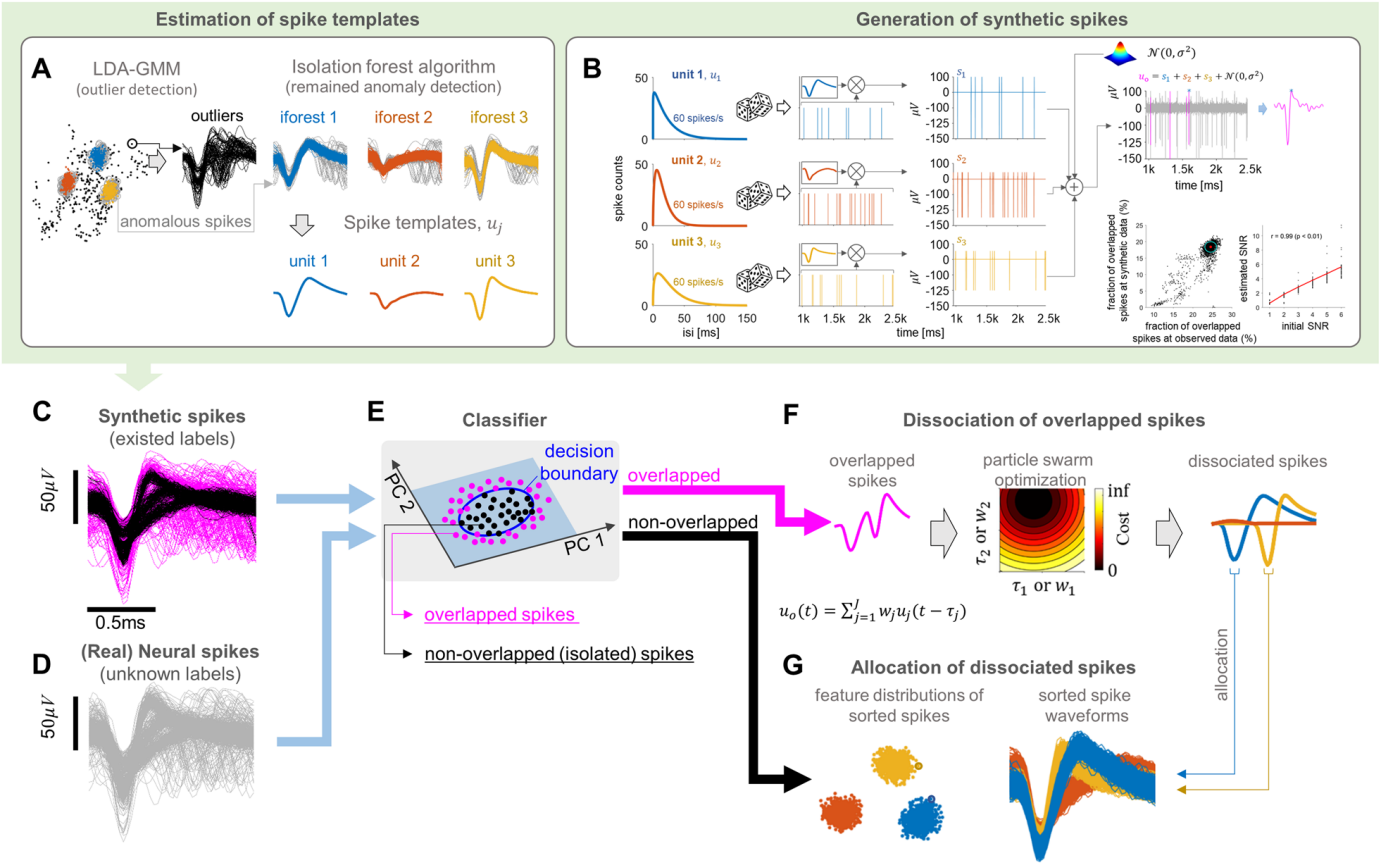
Illustration of the process for identifying overlapping spikes through synthetic data-driven real spike classification. **A** Spike templates can be estimated by taking the sample-wise median of the single unit waveforms isolated by LDA–GMM, and then removing residual anomalies using the isolation forest algorithm. **B** The generation of synthetic spikes proceeds through the following steps: (1) random sampling of interspike intervals (ISIs) following a gamma distribution and cumulative summation to create spike trains. (2) Convolution of spike trains with spike templates, performed independently for each spike template and then summed. (3) Addition of Gaussian white noise. In the generated synthetic spike data, the solid magenta lines represent overlapping spikes (indicated on the far right). The far-right bottom panels represent changes in the fraction of overlapped spikes in the synthetic data depending on the fraction of overlapped spikes in the observed data, and changes in the estimated SNR as the initial SNR changes, respectively. **C**, **D** Real or synthetic spike waveforms obtained by the spike detection. **E** Synthetic data-driven classification to identify overlapping spikes from real data. Blue coloured contour represents the decision boundary determined by SVM. Magenta dots correspond to overlapping spikes, while dark dots denote non-overlapping spikes. **G** Identified non-overlapping spikes can be sorted using common spike sorting methods, such as LDA–GMM, **F** while overlapping spikes can either be removed or broken down into single units. In this study, they were decomposed using the particle swarm optimization algorithm, which is a type of heuristic optimization algorithm

In this paper, we introduce a novel approach for identifying and decomposing overlapping spikes from detected signals, thereby isolating them into single units. Initially, spike snippets (waveforms) are detected by threshold crossing of band-pass filtered extracellular activity. Next, the optimal number of single units and their respective clusters is determined through subspace estimation from the detected snippets using LDA–GMM. An isolation forest algorithm (iForest) is then applied to identify and eliminate anomalous spikes that may perturb spike template estimation within each single unit cluster (see Fig. 2A) [24]. The spike templates are obtained by taking the sample-wise median of the spike waveforms included in their single unit clusters. These spike templates serve for a dual purpose: generating synthetic extracellular activity (see Fig. 2B, C) and decomposing overlapping spikes into single unit spikes using a heuristic optimization algorithm (see Fig. 2F).

The synthetic extracellular activity, which provides spike waveforms and labels for arbitrarily overlapped spikes, is employed to train a classifier for identifying overlapping spikes among the detected ones (see Fig. 2C, E). This arises from the fact that the label information for overlapping spikes cannot be directly obtained from real data. Based on the trained classifier, overlapping spikes are identified from real observed data (see Fig. 2D). These overlapping spikes are often either excluded from analysis or separated into individual single units. Here, we constructed an objective function based on the overlapping spike model (see Fig. 1A) and decomposed it into individual single units using a particle swarm optimization (PSO) algorithm (see Fig. 2F) [25].

To examine the effects of our proposed approach, we primarily utilized simulation datasets through synthetic spike generation procedures rather than real spikes (see Fig. 2A, B). The reason for this choice is that synthetic data provides a definitive ground truth, including clear labels for overlapping spikes, which enables a more controlled and comprehensive evaluation of the proposed method. Therefore, we constructed a synthetic spike generation pipeline based on known ground truth waveforms and interspike interval (ISI) distributions, modeled by a gamma distribution estimated from real neural recordings (as shown in Fig. 2B and described in “Data generation for simulation” section).

While the primary focus was on synthetic datasets, for comparison purposes, we briefly applied the method to a real spike dataset. However, it is important to note that this real dataset was used for supplementary testing, rather than for the main performance evaluation. Our key metrics—recall, precision, and F1 score—were derived mainly from synthetic spike data to investigate how well the method can identify overlapping spikes under controlled conditions. To further evaluate the performance of the proposed method, considering the sensitivity of neural signals to noise, we adjusted the SNR values of the synthesized spikes and analyzed how this affected the classification accuracy of overlapping spikes. Additionally, we explored the decomposition of overlapping spikes and assessed its impact on isolated single-unit spikes.

## Methods

### Data generation for simulation

The synthetic spike generation procedure can serve two purposes: firstly, to provide simulation data for examining the effects of the approach proposed in this study, and secondly, to provide training data for learning a classifier to identify overlapping spikes.

To generate the simulation data, we first assumed that raw extracellular signals would be recorded through a single electrode at a sampling rate of 30 kHz, with a measurement duration limited to 60 s. We initially set the number of neurons to three, assuming that when overlapping spikes occur near this electrode, at least two single units would fire spikes simultaneously or within a short delay. Additionally, it was assumed that the amplitude of each neuron’s spike waveform would decrease according to an inverse-square law with increasing distance from the electrode [26, 27]. In other words, we determined the magnitude based on the inverse-square law, which is expressed as:

1 $${I}_{j}=\frac{{I}_{0,j}}{{\left(1+{d}_{j}\right)}^{2}}$$

where *I*_*j*_ denotes the magnitude of the *j*-th neuron’s spike waveform, *d*_*j*_ is the distance from the electrode tip to neuron *j*, and *I*_*0,j*_ is the magnitude when *d* = 0. The magnitude was set to *I*_*0*_ = 120 μV when fully contacting the electrode tip, based on the actual action potential morphology of the extracellular recordings. Initially, the distances between neurons and electrodes were randomly sampled between 20 and 60 µm to align with the number of spike templates. These distances are then randomly shuffled in each simulation and applied element-wise as weights to the spike templates, which have been rescaled between 0 and 1. Here, we ensured that at least two neurons were placed a short distance apart to reproduce overlapping spikes. Furthermore, we assumed that the ISI of the spike train firing from each neuron strictly followed a gamma distribution [28, 29].

Spike templates, representing the action potential of an isolated single unit, are necessary to generate plausible synthetic spike trains (as detailed in “Spike template estimation” section). To support this, we postulated that the spike templates would be the action potentials of single units, detectable around the electrode tip. In this study, initial spike templates were obtained from a real dataset (see “Real data acquisition” section). These spike templates were convolved with the spike timings to generate simulated spike trains, assuming the firing activities of an ideally isolated single unit. Since the ISIs of real neuronal spike timing are known to follow a gamma distribution, we generate the gamma random numbers to represent the ISIs. The shape and scale parameters of the gamma distribution define the spike firing rate, *f*_*j*_, using the following equation:

2 $${f}_{j}=\frac{1}{{\alpha }_{j}{\theta }_{j}}$$

where *α*_*j*_ represents the shape parameter and *θ*_*j*_ represents the scale parameter of the single unit *j*. Considering the refractory period for each single unit, we initialized *α* with a randomly sampled value from a uniform distribution between 1.01 and 2 [1]. The firing rates were set to 60 Hz to ensure that the probability of overlapping spikes occurring exceeded 20%, resulting in each *θ* being approximately 0.05 (see Figure S1 in Supplementary Material). Note that the firing rate was set uniformly to ensure a consistent proportion of overlapping spikes among all detected spikes (see Figure S2 in Supplementary Material).

We generate random numbers representing ISIs following the gamma distribution based on these parameters. In this process, we use the “gamrnd” function from the Matlab toolbox. Following the ISI generation, samples within the 48-point refractory period are excluded, reflecting the physiological phenomenon where subsequent spikes do not occur within 2.5 ms in the same neuron [1]. The spike trains are then created by taking the cumulative sum of the ISI samples. Spike templates are convolved with the spike trains randomly generated as the number of spike templates individually.

These ideally isolated single unit activities are then summed across multiple spike templates. This allows us to access information regarding the occurrence of overlapping spikes and the unit labels involved. Finally, Gaussian white noise with a signal-to-noise ratio (SNR) of a specific range (from 1 to 3 detailed in “Assessment” section) is applied. The SNR is defined as the minimum peak-to-peak spike waveform scale relative to the root mean square of the spike-free noise segment, as detailed in [30]. Note that in this study, we did not consider background signal drift.

### Real data acquisition

A real dataset was obtained by chronically implanting a 96-channel microelectrode array into the primary motor cortex (M1) of one Rhesus macaque. Spontaneous neural activities were recorded while the monkey freely moved its arm without performing any task instruction. In this study, we evaluated the data obtained from only channels in which distinct neural spikes were observed. Neural signals were acquired using a Cerebus system (Blackrock Neurotech, Salt Lake City, UT) for a duration of 180 s at a sampling rate of 30 kHz.

### Band-pass filtering and spike detection

The preprocessing procedures for extracellular activity in our proposed approach follow the traditional spike sorting pipeline. We constructed a 6th-order Butterworth filter for band-pass filtering. Specifically, we filtered the raw extracellular signals through a 300–3000 Hz band-pass filter. Subsequently, for spike detection, we employed the method proposed by Quiroga et al. [31]. According to their method, the threshold, thr, is determined based on the standard deviation of the background noise of the filtered signal, **x**, as defined in Eq. 3.

3 $$thr=c\cdot \text{median}\left(\frac{\left|\mathbf{x}\right|}{0.6745}\right)$$

where the denominator, 0.6745, is derived from the inverse of the cumulative distribution function for the Gaussian distribution, and *c* is the constant reflecting spike detection sensitivity, which can be calculated as std(**x**)/median(|**x**|). After the threshold was crossed, we stored the putative spike waveforms, U = {**u**_1_, **u**_2_, …, **u**_M_} ∈ ℝ^44×*M*^, with 44 samples (equal to 1.5 ms) for each detected spike, where *M* denotes the number of detected spikes. We aligned spike waveforms based on their minimum peaks, with 10 samples preceding and 34 samples following its peak latency.

### Spike template estimation

To estimate the spike template, we implemented a two-step procedure involving LDA–GMM and iForest. LDA–GMM is an iterative subspace learning method that combines LDA and GMM clustering [22]. In simple terms, it iteratively updates the discriminant prediction matrix using GMM until achieving maximum cluster separability. The objective function to be maximized can be expressed as follows:

4 $$\underset{{\text{W}}^{*}{\text{L}}^{*}}{\text{max}}Trace\left(\frac{{\text{W}}^{T}{\text{S}}_{b}\text{W}}{{\text{W}}^{T}{\text{S}}_{W}\text{W}}\right)$$

where L represents the labels clustered by GMM, and S_*b*_ and S_*W*_ are the covariance matrices representing the between-class and within-class variances, respectively. The superscript *T* denotes the transpose of the matrix. Thus, this algorithm can identify single unit spike candidates by detecting outliers. However, the efficacy of outlier detection may be affected by the initialization of GMM, owing to the intricate multimodal distribution of data within the subspace. Therefore, we additionally applied the iForest, which is one of the unsupervised anomaly detection methods, to potentially find spike templates close to single unit spikes, further enhancing the quality of the clustered spike waveforms. The iForest constructs an ensemble of isolation trees (iTrees) from the datasets, identifying anomalies as instances with shorter average path lengths in the iTrees [24]. With path lengths, h(**u**), anomaly score, *s*_*a*_, reflecting the degree of anomaly is given as follows:

5 $${s}_{a}\left(\mathbf{u},n\right)={2}^{-\frac{E\left(\text{h}\left(\mathbf{u}\right)\right)}{c(n)}}$$

where *E*(h(**u**)) is the average of h(**u**) for collected isolation trees, and *c*(*n*) is the normalizing factor, which can be described as the average of h(**u**) given node *n*. The outlier score ranges between 0 and 1, with values closer to 1 indicating a higher likelihood of being an outlier.

Initially, we differentiated each detected spike waveform with respect to time (*du*/*dt*), followed by the application of LDA–GMM [22]. This approach was chosen because it is well-known that differentiated waveforms tend to outperform undifferentiated ones [22]. Each clustered waveform group was refined, by detecting and eliminating anormalies through the iForest. At this point, we optimized the contamination fraction of the iForest by calculating the unimodality of the feature distribution through four-fold cross-validation for each waveform group. The spike templates were then estimated by taking the median of the waveforms for each waveform group (see Fig. 2E).

The number of spike templates confirmed from synthetic spikes can be equal to or fewer than the predefined number (three units in this simulation) during the generation of synthetic spike data, depending on the specified changes in SNR. However, to estimate spike templates from real neural spikes, we needed to determine the number of potentially valid clusters (to be used as spike templates). We employed the LDA–GMM method, gradually increasing the number of clusters (from 2 to 5) according to the method proposed by Keshtkaran and Yang [22]. Subsequently, we examined whether the data distribution in the subspace was over-clustered using the Anderson–Darling test. This method aligns with optimizing the contamination fraction of the iForest. All these procedures were implemented based on the “iforest” function from the Matlab toolbox and the LDA–GMM toolbox available in [22].

### Synthetic spike generation for classifier

When building a classifier, it is crucial to reconstruct synthetic spike data (or simulation data) with characteristics as similar as possible to the observed data. Initially, we estimated the parameters of the gamma distribution representing the ISI distribution of the observed data using maximum likelihood estimation, performed using the “gamfit” function in the Matlab toolbox. However, if the SNR is low, fewer spikes may be detected, potentially leading to fitting failure. So, in this scenario, we randomly sampled alpha between 1.01 and 2, the same as the simulation data generation process (see “Data generation for simulation” section). By utilizing the estimated parameters of the gamma distribution, we estimated the firing rate according to Eq. 2. Using these parameters of the gamma distribution and the spike templates estimated from the observed data, we generated synthetic spike data following the method mentioned in “Data generation for simulation” section. To minimize heterogeneity with the observed data, the proportion of overlapping spikes in the synthetic spike data was maintained to be similar to those of the observed data, and the SNR was set to the SNR estimated from the observed data.

### Classification of overlapping spikes

To classify overlapping spikes from detected spikes, ground truth is necessary to train the classifier. In this section, we detail constructing the classifier using synthetic spikes to identify overlapping spikes and its testing with real detected neural spikes.

### Feature extraction

Projecting detected spikes into a low-dimensional subspace is beneficial as it efficiently summarizes essential information. For classifier training, we first projected the detected spikes into a low-dimensional space using principal component analysis, which can be expressed as:

6 $$\mathbf{z}={\text{C}}^{T}\overline{\mathbf{u} }$$

where **z** is the score of the principal components, C^*T*^ ∈ ℝ^*D*×*M*^ denotes the transposed loading matrix, and $\overline{\mathbf{u} }$ is the centralized waveforms. We determined dimensionality, *D*, by identifying the minimum number of components needed to account for at least 90% of the data variance. This determination was based on the eigenvalues’ contribution the total variance of the training data, which on average results in *D* being 15. To ensure dimensional consistency across both datasets, it is important to perform subspace learning when the SNR and single unit waveforms of synthetic and real neural spikes are similar.

### Classification

Since the principal component scores for synthetic spikes with the label information of overlapping spikes exhibit inherently non-linear class distributions, we opted to construct a support vector machine (SVM) with a radial basis function (RBF) kernel [32]. We set the box constraint to 1, a value optimized to achieve the highest possible classification accuracy.

### Particle swarm optimization algorithm

Based on the model illustrated in Fig. 1, the process of decomposing overlapping spikes requires the optimization of delay time, *τ*, and coefficients (also known as contribution index), *w*, for each single unit *j*. The objective function, aimed at minimizing the mean absolute error of this model, is formulated as:

7 $$\underset{{\omega }^{*}{\tau }^{*}}{\text{argmin}}\frac{1}{44}\left|\sum_{t=1}^{44}\sum_{j=1}^{J}{\omega }_{j}{u}_{j}\left(t-{\tau }_{j}\right)-{u}_{0}\left(t\right)\right|$$

where *J* denotes the total number of the spike templates, *u*_*o*_ is the observed overlapping spike. If the number of single units is two, overlapping spikes could be modelled as simple combinations based on time delay changes and partitioned using cross-correlation, etc. However, as the number of combinations increases exponentially with the number of single units, we estimated the variables {time delay (*τ*) and coefficients (*w*)} of the overlapping spike model using Particle Swarm Optimization (PSO), a metaheuristic search algorithm that iteratively explores the solution space [25]. PSO operates based on a set of candidate solutions, called particles, and optimizes the solution by adjusting the particles’ positions and velocities within the search space. In each iteration, PSO evaluates the movement of each particle to find a better position, which is considered the optimal solution. The estimated variables include *τ* and *w*, each of which is constrained within a certain range. Specifically, we limited the *τ* to be between − 44 and 44, and w between 0 and 1, depending on the number of estimated spike templates. To prevent excessive exploration of the search space, the inertia weight damping ratio was set as 1. Additionally, to limit the movement of particles in each iteration, the inertia weight, *w*_*χ*_, was set as 0.55. Additionally, the number of PSO particles was fixed at 100, and the algorithm was repeated 1000 times for each spike. This represents the experimental number of iterations in which the objective function can be minimized. If the positions (or variables) are no longer updated, the iteration stops.

### Assessment

We evaluated the proposed method’ sensitivity to noise by changing SNR from 1 to 3 with 0.1 interval. To assess the impact of noise, we iteratively regenerated simulation data up to 1000 times. We then compared the proposed method with the following approaches: “outlier detection using LDA–GMM (LG)”, “outlier detection using iForest + LDA–GMM (IF^+^)”, and “testing the subspace scores of observed spikes using a synthetic spike subspace score-based classifier with PCA {PCA(syn → obs)}”.

To determine iForest’s effectiveness on template spikes, we calculated the root-mean square error (RMSE) between the refined and the ground-truth spike waveforms and compared it to the distribution of all spike errors without iForest. This can be expressed in the following formula:

8 $$RMS{E}_{j}=\sqrt{\frac{1}{M}\sum_{m=1}^{M}{\left({\mathbf{u}}_{j}-{\widehat{\mathbf{u}}}_{m,j}\right)}^{2}}$$

where ${\widehat{\mathbf{u}}}_{m,j}$ denotes the refined spike waveform of the m–th spike within the single unit j. Based on this, we quantified the relative iForest effect by defining it as the difference in RMSE between IF^+^ and LDA–GMM. The iForest effect is expressed as follows:

9 $$\text{iForestEffect}=\frac{{\text{IF}}_{\text{RMSE}}^{+}-{\text{LG}}_{\text{RMSE}}}{{\text{IF}}_{\text{RMSE}}^{+}+{\text{LG}}_{\text{RMSE}}}$$

where ${\text{IF}}_{\text{RMSE}}^{+}$ and ${\text{LG}}_{\text{RMSE}}$ denote the RMSE of LG and IF^+^, respectively. This effect was represented as a function of SNR.

In addition, since overlapping spikes occur in about 20% of total spikes (see Figure S1 in Supplementary Material), we quantified precision, which represents the ratio of predicted overlapping spikes among actual overlapping spikes. Additionally, recall, which indicates the probability that the predicted overlapping spike contains an actual overlapping spike, and the F1-score, which quantifies performance by considering the trade-off between precision and recall, were also calculated. Statistical tests for classification performances were conducted using the Wilcoxon rank sum test and Kruskal–Wallis test, with a Tukey–Kramer correction applied for the post-hoc multiple comparison testing. In addition, we compared the effects of the proposed approach, and LDA–GMM across varying SNRs using the Friedman test.

To evaluate the decomposition of overlapping spikes, we calculated cluster-wise precision, recall, and F1-score between true spikes and those from reallocated spike trains of decomposed single units. We also confirmed the correlation between the time lags of actual spikes and their estimated counterparts. As matching the stochastic ISI distribution is crucial for assessment, we calculated the absolute difference between Gamma distribution parameters estimated from actual and estimated spike trains. In addition, we compared the proposed method to LDA–GMM by measuring event synchronization between single unit spike trains. Event synchronization is quantified by the number of nearly simultaneous occurrences of spike events, based on the relative timings of events within the time series, such as local maxima. We implemented this metric with Matlab toolbox for Event Synchronization, which is available at the [33, 34].

For real neural spikes, we have limited access to ground truth data. We thus perform a qualitative comparison by examining the change in ISI distribution of overlapping spikes. In the verification of the real neural data, we estimated and compared the Gamma distribution parameters of the ISI distribution to the results of LDA–GMM, by referencing the refractory periods of neural spike trains.

## Results

### Estimation of spike templates

**Fig. 3:**
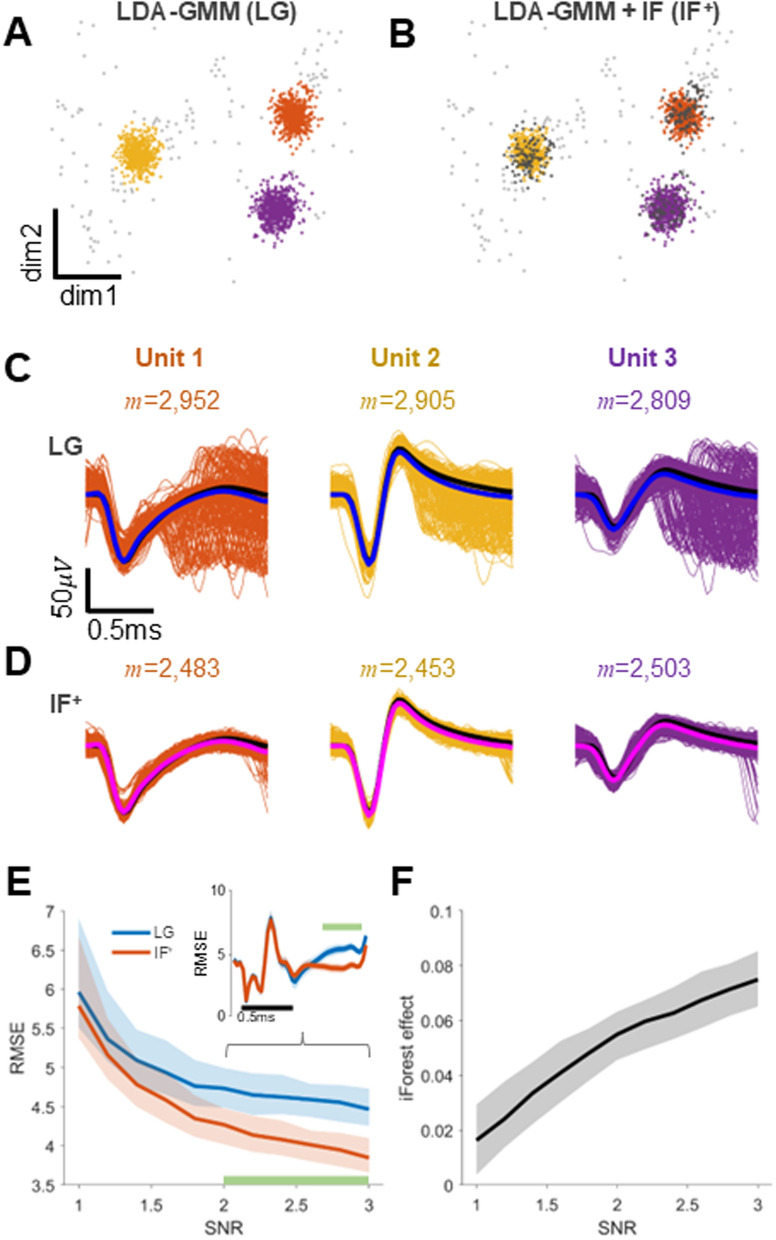
The effects of the combination of LDA–GMM and iForest algorithm on finding spike templates. **A**, **B** Visualization of feature spaces for LG and IF^+^. Grey dots denote outliers, including overlapping spikes, detected by only LDA–GMM, and the dark gray dots indicate anomalies additionally detected by iForest. Other colors (orange, yellow, and purple) dots denote the single units. **C** Single unit waveforms corresponding to LG in **A**. Each column corresponds to each single unit. Solid blue lines denote spike templates obtained by LG. Solid dark line represent the true spike waveforms. The *m* represents the number of spikes. **D** Single unit waveforms corresponding to IF^+^. Solid magenta lines denote spike templates obtained by IF^+^, the rest is the same as in **C**. **E** RMSEs between true and estimated spike templates in the SNR conditions. The inset shows changes in RMSE over time, averaged for SNR > 2. Blue solid line corresponds LG, the orange solid line denotes IF^+^. The light-green solid line at the bottom of the panel indicates a significant difference (*p* < 0.05, Wilcoxon rank sum test). **F** iForest effects in SNR condition changes, which denotes the difference between the RMSEs of LG and those of IF^+^

We initially investigated the synergistic impact of combining LDA–GMM and iForest on spike template estimation. Figure 3 illustrates the performance comparison between LG (Fig. 3A) and IF^+^ (Fig. 3B), utilizing simulation data derived from three distinct single unit models. IF^+^, integrating LDA–GMM and iForest, was effective at identifying outliers surrounding the unit clusters and detecting anomalous spikes inside the cluster. In particular, we were able to remove spikes suspected to be overlapping spikes and estimate a waveform close to the real spike of a single unit through the average of the refined waveforms (Fig. 3C, D). Figure 3E shows the RMSE of the real and estimated spike waveforms according to changes in SNR of each unit for each method. Although the spike templates estimated by each method appeared visually similar in mimicking the actual spike waveform, the IF^+^ exhibited a statistically significantly lower RMSE compared to LG, particularly when the SNR ≥ 2 (*p* < 0.01, according to the Wilcoxon rank sum test). Under these conditions, the iForest effect showed a strong linear correlation with SNR increases (*r*^2^ = 0.98, *p* < 0.01, F-value = 7056.6), confirmed by a linear regression analysis between SNR and the iForest effect across repeated trials (see Fig. 3F). We also measured cosine similarity to assess the match between spike templates estimated by each method and the true spike waveforms. For SNR values ≥ 2, IF^+^ scored 0.19 higher than LG (*p* < 0.05).

### Classification of overlapping spikes

We examined the impact of implementing the proposed method on the training of the overlapping spike classifier, following the generation of synthetic data using the estimated spike templates. In this context, we considered the effects of IF^+^ as well, since not only outliers detected by LDA–GMM but also their anomalies detected by iForest are likely to be overlapping spikes. All comparative analysis was rigorously and repeatedly conducted by dividing them into training and testing datasets for all SNR conditions.

**Fig. 4:**
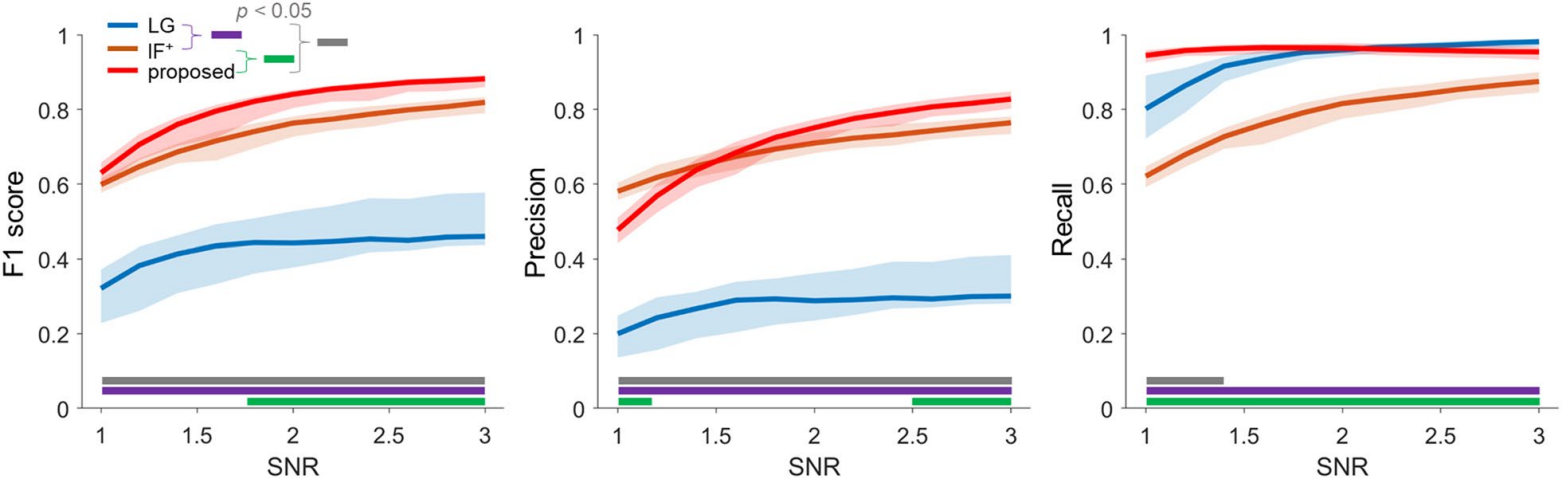
Comparison of the classification performance for identifying overlapping spikes. The F1 score, precision, and recall are displayed from left to right. Each solid line represents the median performance of each method for identifying overlapping spikes. Shaded regions denote 25% and 75% of the measurement distribution. The purple, green, and gray solid lines at the bottom of the panel indicates a significant difference (*p* < 0.05, Kruskal–Wallis test)

The left panel in Fig. 4 illustrates the changes in F1 score as a function of SNR variations. For each SNR ≥ 1.8, the proposed method demonstrated a significantly higher F1 score (*p* < 0.01, Kruskal–Wallis test, post hoc analysis for multiple comparison testing with a Tukey–Kramer correction) compared to both IF^+^ and LG. At an SNR < 1.8, however, the proposed method did not significantly differ from IF^+^ (*p* = 0.43) but was significantly higher compared to LG (*p* < 0.01). F1 score of IF^+^ was a higher F1 score than that of LG for all SNR conditions (*p* < 0.01). Based on these results, we performed a Friedman test to compare methods while accounting for the effects of factors related to changes in SNR. All methods showed significance for F1 score (*p* < 0.01), with a calculated χ^2^ = 697.2. A post hoc analysis for multiple comparison test with a Tukey–Kramer correction revealed that the proposed method had the highest performance (*p* < 0.01).

The middle panel of Fig. 4 illustrates the precision. The proposed method yielded a significantly higher precision at SNR > 2.5 (*p* < 0.01, Kruskal–Wallis test, post hoc analysis with Tukey–Kramer correction) compared to both IF^+^ and LG. Meanwhile, the proposed method showed a lower precision compared to IF^+^ when SNR < 1.25 (*p* < 0.01). Both the proposed method and IF^+^ yielded a higher precision than that of LG for all SNR conditions (SNR ≥ 1: *p* < 0.01). The Friedman test for all SNR conditions produced χ^2^ = 628.3, and was significant for each of the methods (*p* < 0.01). A post hoc analysis using Tukey–Kramer correction revealed that the proposed method had significantly higher precision relative to both IF^+^ and LG (*p* < 0.01).

**Fig. 5:**
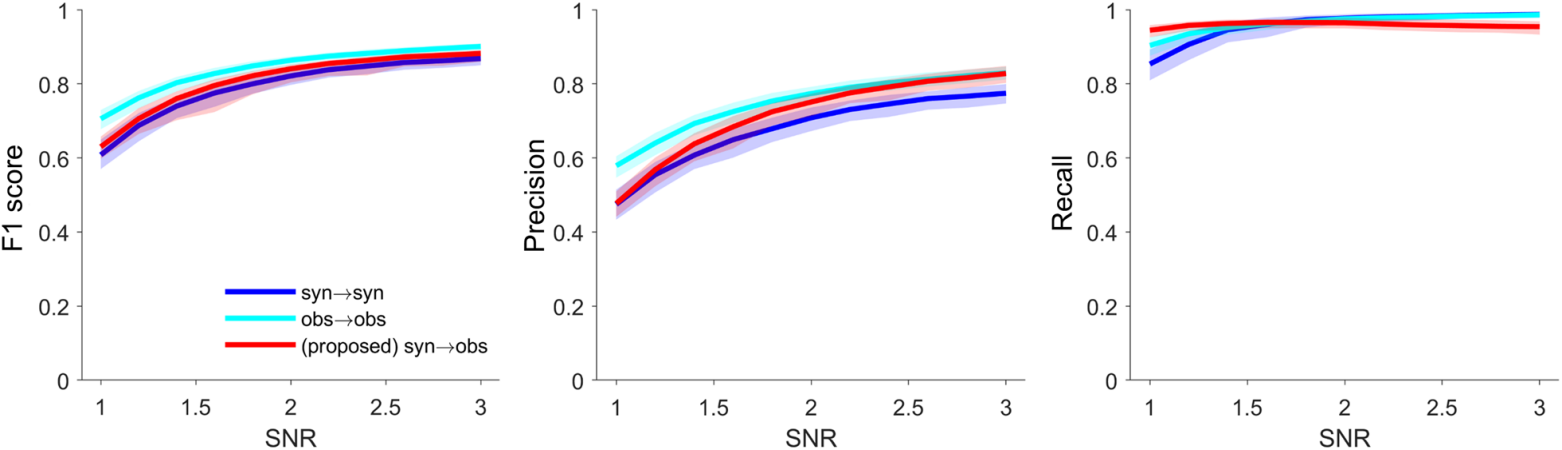
Comparison of synthetic and observed data sets’ combinations. The F1 score, precision, and recall are displayed from left to right. Each solid line represents the median performance of each method for identifying overlapping spikes. Shaded regions denote 25% and 75% of the measurement distribution. The “(proposed) syn → obs” is consistent with the “proposed” in Fig. 4

Recall, shown in the right panel of Fig. 4, indicates that the proposed method does not significantly differ from LG when SNR > 1.38. However, the proposed method was maintained at a consistently higher level compared to IF^+^ and LG for all SNR conditions. Meanwhile, IF^+^ yielded a significantly lower recall compared to both the proposed method and LG. The Friedman test yielded χ^2^ = 712.2, showing significance for each of the methods (*p* < 0.01). A post hoc analysis using Tukey–Kramer correction revealed no significant difference between the proposed method and LG (*p* = 0.47) for all SNR conditions, while the proposed method and LG showed significant differences with IF^+^ (*p* < 0.01). We also examined the effects of the classifier’ self- calibration on synthetic “syn → syn” and observed (for test) datasets “obs → obs” with to verify whether the results of the proposed method are overfitting through a fourfold cross validation (see Fig. 5). If the performance of the proposed method yields a significantly lower than that of both “syn → syn” and “obs → obs”, then it is likely to be overfitted. This is because if the generative conditions of spike trains change, the properties of the synthetic and observed datasets may become disparate, potentially leading to a degradation in the performance of “syn → obs” instead. Figure 5 shows that the proposed method produced a similar performance for all SNR conditions. The Friedman test yielded χ^2^ = 532.6, showing signfiicance for each of the combinations (*p* < 0.01). A post hoc analysis using Tukey–Kramer correction revealed not sigifiicant difference among all combinations {“obs → obs” vs. the proposed method: *p* = 0.85, “syn → syn” vs. the proposed method: *p* = 0.91, and “syn → syn” vs. “obs → obs”: *p* = 0.32}.

### Decomposition of overlapping spikes

**Fig. 6:**
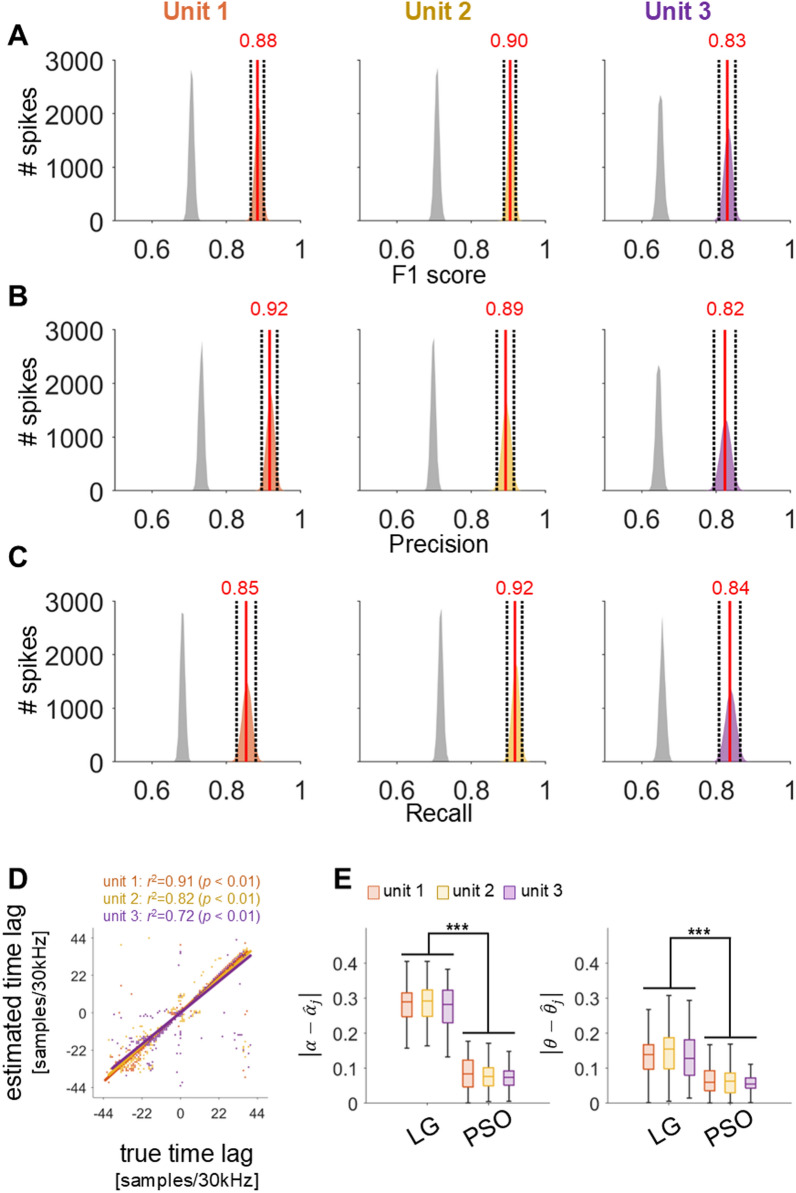
Decomposition of overlapping spikes. **A**–**C** Identification performance (F1 score, precision, and recall) between true single units and dissociated single units. Filled grey plots denote the distribution of the shuffled data and filled orange, yellow, and purple plots represent the distribution of performance for each single unit. Solid red vertical line denotes the median and dashed dark vertical lines correspond to 25% and 75% of their distribution. Red coloured numeric above the vertical lines denotes the median value. **D** The correlation between true and estimated time-lags. Each dot indicates the individual spikes, and each coloured solid line represents the trend line fitted by the linear regression. **E** Comparison of the absolute errors between true and estimated gamma parameters, *α* and *θ*. Asterisks denote significant difference (***p < 0.001, Friedman test, post hoc analysis with multiple comparison based on a Tukey–Kramer correction)

We can either exclude identified overlapping spikes from our analysis or decompose them into single units to enrich the neural information. We used the PSO to decompose overlapping spikes into single units based on the objective function of the overlapping spike model shown in Fig. 1. Here, spike generation and its decomposition processes were repeated 500 times. Figure 6A illustrates the F1 scores comparing decomposed single units derived from overlapping spikes with the ground truth. Significance levels were determined via 10,000 non-replacement samplings, presenting the following quantiles for each unit: 25% quantile = 0.85, median (50%) = 0.86, 75% quantile = 0.87 for unit 1; 25% = 0.91, 50% = 0.92, 75% = 0.92 for unit 2; 25% = 0.76, 50% = 0.77, 75% = 0.78 for unit 3 in Fig. 6A. Each decomposed unit significantly matched its corresponding actual unit in terms of precision {25% = 0.87, 50% = 0.87, 75% = 0.88 for unit 1; 25% = 0.92, 50% = 0.93, 75% = 0.93 for unit 2; 25% = 0.75, 50% = 0.76, 75% = 0.77 for unit 3 in Fig. 6B} and recall {25% = 0.84, 50% = 0.85, 75% = 0.86 for unit 1; 25% = 0.90, 50% = 0.91, 75% = 0.92 for unit 2; similar results were obtained for 25% = 0.78, 50% = 0.79, 75% = 0.80 for unit 3 in Fig. 6C}. Figure 6D illustrates the correlation between the time-lag of the actual single unit and the decomposed single unit, showing a strong positive correlation for each single unit, with a relatively high error variance observed for unit 3 {42.6, 31.3, 78.3 for each unit}. Further post hoc analysis revealed significant differences among all combinations of methods. The shape parameters estimated by each method and the ground truth are as follows: LG: 1.63, 1.73, 0.87 for each unit; PSO: 0.17, 0.16, 0.47 for each unit; ground truth: 0.81, 0.77, 0.84 for each unit. Meanwhile, for the scale parameter θ, PSO exhibited a relatively lower error rate compared to LG (*p* < 0.01, χ^2^ = 5,156, Friedman test). Further post hoc analysis revealed significant differences among all combinations of methods. The scale parameters for each method and the ground truth are as follows: LG: 0.01, 0.01, 0.23 for each unit; PSO: 0.09, 0.09, 0.08 for each unit; ground truth: 0.02, 0.02, 0.02 for each unit.

**Fig. 7:**
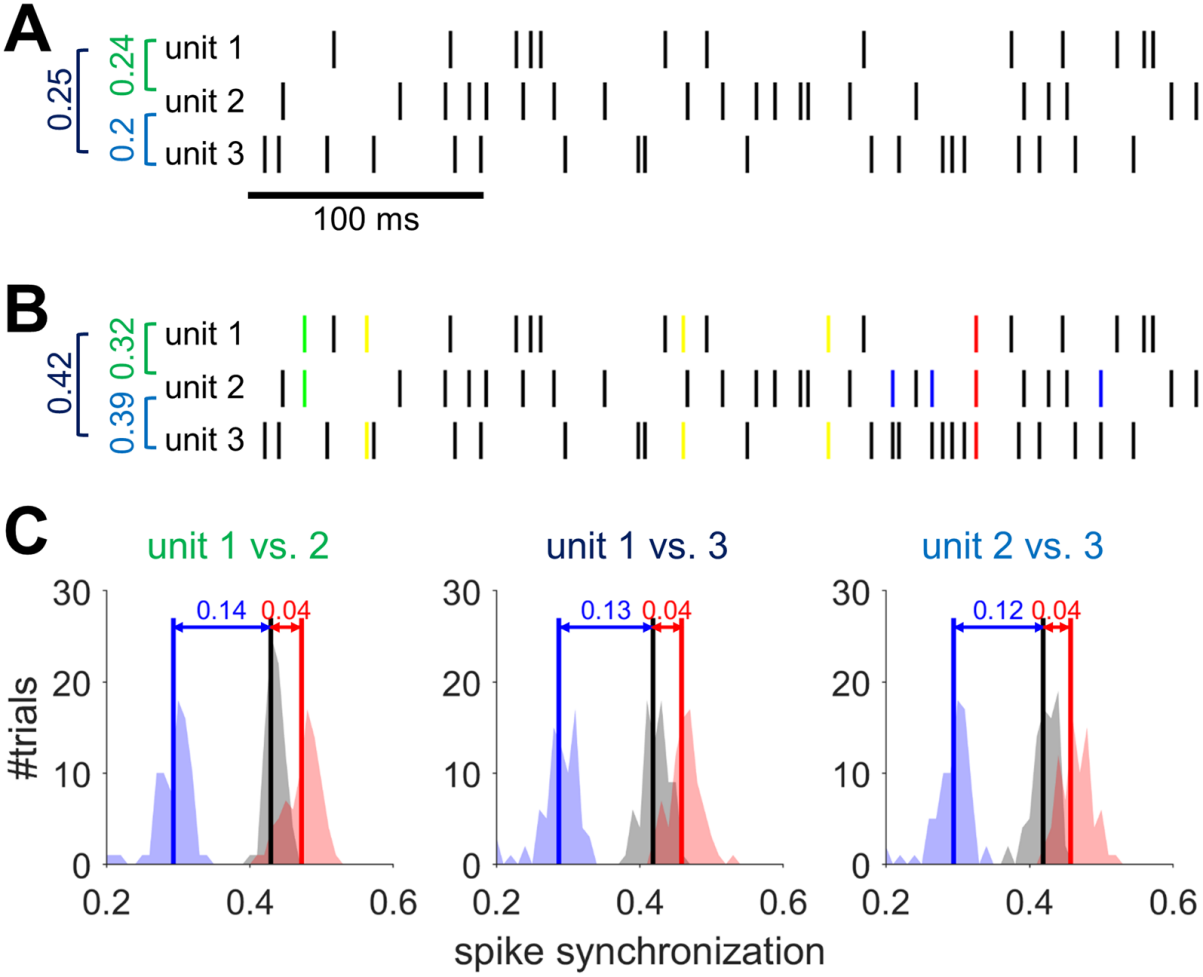
Comparison of spike train synchronization with and without overlapping spike reallocation. **A** Examples of spike trains after applying LG, excluding overlapping spikes. The dark vertical lines denote spike. The numeric values on the left represent the spike synchronization index between units enclosed in square brackets. **B** Examples of spike trains with reassigned decomposed overlapping spikes. The coloured vertical lines denote single units’ spikes decomposed from overlapping spikes. The rest is the same as in **A**. **C** The distribution of spike synchronization indices for each pair of units. The shaded blue patch and blue vertical line represents the distribution and median of the synchronization indices between spike trains across repetitions of 500 times, excluding overlapping spikes, after applying LG. The shaded red patch and red vertical line denotes the distribution and median of the synchronization indices between spike trains, reassigning decomposed overlapping spikes, after applying the proposed method. The shaded grey patch and dark vertical line represent the distribution and median of the synchronization indices between actual spike trains of single units. The numeric value at the top of the distribution indicates the absolute difference of the median for each distribution

Additionally, we conducted comparative analysis between the spike trains reallocated the decomposed overlapping spikes into corresponding clusters and the spike trains excluded the overlapping spikes, by measuring spike synchronization between sorted single units (see Fig. 7). Figure 7A shows examples of spike trains excluding times considered as overlapping spikes. Figure 7B depicts examples of spike trains reallocating times of decomposed overlapping spikes into single unit spikes from equal datasets. The spike synchronization distribution of PSO closely resembled that of the actual spike trains for all unit combinations. The median difference between unit 1 and 2 was 0.1 (*p* < 0.01, Wilcoxon rank sum test). Similarly, between unit 1 and 3, the median difference was 0.09 (*p* < 0.01), and between unit 2 and 3, it was 0.08 (*p* < 0.01) (see Fig. 7C).

### Effects on real data

**Fig. 8:**
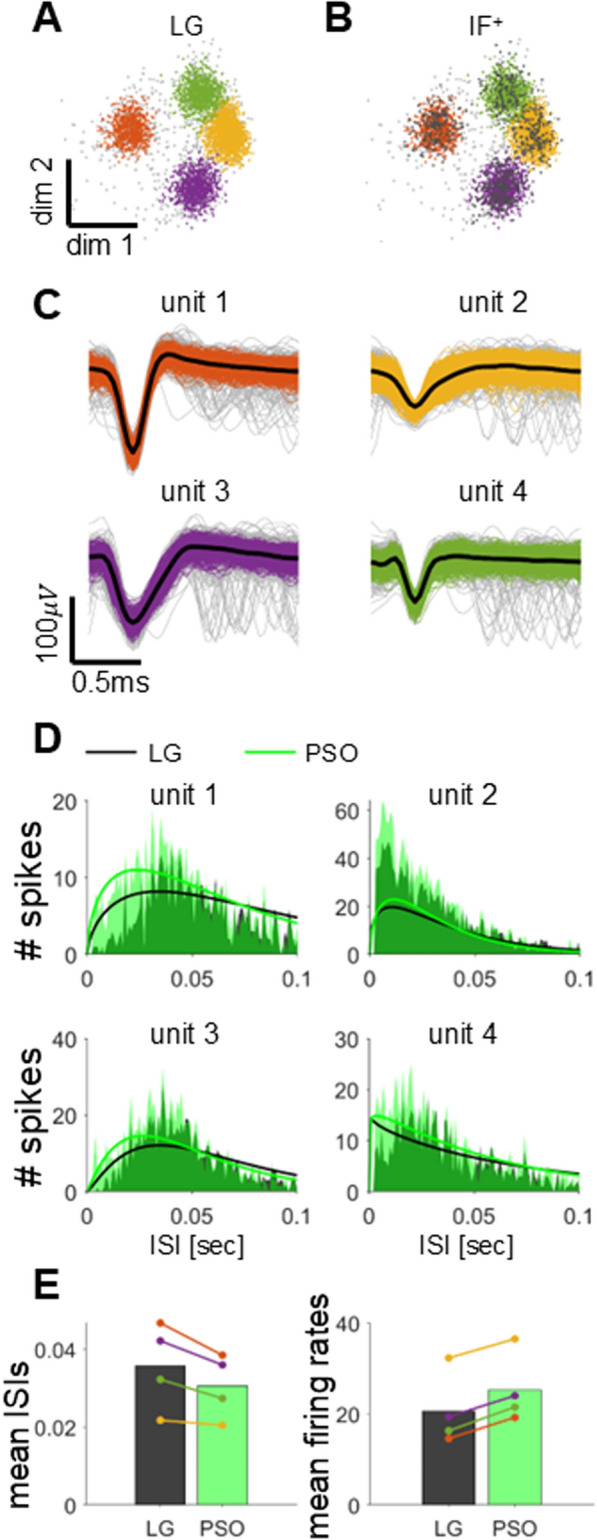
Effects of the proposed method on real data. **A** Feature distribution for LG. Light-grey dots represent outliers, and orange, yellow, purple, and green dots denote isolated single units, respectively. **B** Feature distribution for IF^+^. Data and all formats are the same as **A**. **C** Spike waveforms for single units sorted with IF^+^. The colours of the solid lines are identical to the specifications of **A**. Dark solid lines represent the median of the isolated spike waveforms, and light-gray lines denote anomalies detected by iForest. **D** The ISI distributions of spikes obtained by two methods for each single unit are illustrated, where the green represents PSO, and the dark represents LG. The solid lines corresponding to each colour depict the results of fitting with the gamma function. **E** The left panel represents the differences in the mean ISIs between LG and PSO, and the right panel denotes the difference in the mean firing rates between LG and PSO. Each coloured line corresponds to a single unit

We assessed the effectiveness of our proposed method by analyzing neural signals from the M1 area of rhesus monkeys. Four single units were estimated from this data, and a total of 4794 spikes were detected. Figure 8A illustrates the distribution of features across spike clusters and outliers identified by LG, detailing the spike counts per unit as follows: 879 (17.7%), 1636 (32.9%), 897 (18.0%), 1165 (23.4%), with 217 (8%) of the spikes categorized as outliers. Figure 8B shows the feature distribution of spikes refined using IF^+^, where the number of spikes for each unit was 735 (14.8%), 1466 (29.5%), 759 (15.3%), and 983 (19.8%), respectively, whereas those of outliers was 1031 (20.7%) (see Table 1).

**Table 1:** The identified number of spikes for each isolated unit

	Unit 1	Unit 2	Unit 3	Unit 4	Outliers
LG	735 (14.8%)	1466 (29.5%)	759 (15.3%)	983 (19.8%)	1031 (20.7%)
PSO	873 (18.2%)	1541 (32.1%)	945 (19.7%)	1173 (24.5%)	262 (5.5%)

The waveforms from each spike cluster were averaged, deriving four distinct spike templates, as depicted in Fig. 8C. These spike templates were utilized to generate synthetic spikes with SNRs estimated from the observed data. The observed data was evaluated with a classifier built based on these synthetic spikes to detect redundant spikes. The number of overlapping spikes detected by the proposed method was 739 (15.4%).

Next, we decomposed the identified overlapping spikes using the PSO. We then included the decomposed single units from each overlapping spike in the corresponding cluster, where the number of spikes in each unit was increased by 3.4%, 2.6%, 4.4%, and 4.7%, respectively, where as those of the outliers was decreased by 15.2% (see Table 1).

Figure 8D displays the ISI distributions of single unit spike trains reconstructed through PSO and those reconstructed by LG. Across all neurons, PSO demonstrated a distribution closely similar to the gamma distribution of LG, as shown in Fig. 8D. However, PSO allowed reallocating a greater number of spikes compared to LG, while maintaining their refractory periods. Shape and scale parameters for each unit fitted by the maximum likelihood estimation was {unit 1 = (1.63, 0.05), unit 2 = (1.39, 0.03), unit 3 = (2.25, 0.03), and unit 4 = (0.96, 0.08)}. We also computed the median interspike intervals (ISIs) for four single-unit spike trains reconstructed using PSO and LG. The mean of these median ISIs was 0.03 ± 0.01 for PSO and 0.04 ± 0.01 for LG, with LG being slightly higher by 0.01 ± 0.003. Both parametric (*p* = 0.06, paired t-test) and nonparametric (*p* = 0.48, Wilcoxon rank-sum test) analyses showed no significant difference in the mean ISIs between PSO and LG. However, the mean firing rate for single units was 25.3 ± 7.67 Hz for PSO and 20.7 ± 8.01 Hz for LG, with PSO being 4.65 ± 0.41 Hz higher than LG. While the nonparametric test showed no significant difference in mean firing rates (*p* = 0.34, Wilcoxon rank-sum test), the parametric test revealed a significant difference (*p* < 0.001, paired t-test) (see Fig. 8E).

## Discussion

This study introduces a classifier based on synthetic spikes designed to identify overlapping spikes within neural data, even in the absence of a definitive ground truth. We proposed an approach that can be tested on real observation data through a systematic reconstruction of synthetic data based on the given real data. Initially, single unit spike clusters were estimated from observed data using LDA–GMM, followed by the detection and exclusion of residual outliers using iForest. This method improved the estimation of spike templates closer to the actual spike waveforms of single units by eliminating residual anomalies within the spike clusters in the feature distribution. While there was no significant numerical difference compared to scenarios without iForest, the effect became more pronounced at higher SNR. This is because iForest is adept at detecting outliers associated with a higher rate of overlapping spikes (Figure S1 in Supplementary Material).

Furthermore, we employed synthetic spikes to generate putative spike templates, ensuring that the synthetic spikes resembled the signal characteristics of observed data as closely as possible. Synthetic spikes were used to train a classifier for detecting overlapping spikes in the observed data. However, it is important to note that synthetic spikes might provide several ground truth but could distort feature information based on SNR and spike waveform structure, making them challenging for evaluating real data. We generated synthetic spikes using the described method and trained an SVM based on the ground truth for overlapping spikes. We compared three methods {LG, IF^+^, and the proposed method} under varying SNR conditions. F1 score, precision, and recall were calculated to address imbalanced classes, given the low ratio of overlapping spikes to the total detected spikes. The proposed method exhibited high accuracy in all aspects, and IF^+^ showed a relatively better performance levels compared to LG, possibly due to anomalies that were detected by iForest could significantly correspond to the overlapping spikes within each cluster.

Identified overlapping spikes can be excluded from the analysis or decomposed into single units and added to the sorted spike clusters. We successfully decomposed overlapping spikes using the PSO algorithm, a heuristic optimization algorithm. Note that the key elements of the proposed method include the isolation forest algorithm and the use of synthetic spikes to effectively identify overlapping spikes. While PSO was utilized in this study to explore parameter optimization, it may not be the only or even the optimal approach. Alternative optimization methods could be considered to more effectively identify parameters that explain the overlapping spike phenomena. These alternatives could potentially enhance the accuracy and robustness of spike decomposition. Despite the computational expense of PSO, it effectively estimated time-lag and combinations of decomposed single units, providing valuable analysis opportunities (Figs. 6, 7). Particularly, when the ISI distribution was modeled by including spikes from the decomposed single unit in the sorted spike cluster, it closely resembled the ISI of the ground truth single unit (Fig. 6D). This not only ensures reliable spike sorting but also proves useful in capturing neurons responding to fast stimuli.

Moreover, reassigning spikes from a decomposed single unit allows us to obtain accurate spike synchronization measurements. We measured the spike synchronization between the spike train of the actual single unit and the spike train of the reassigned single unit, demonstrating that reusing the output of overlapping spikes is more beneficial than excluding them. Based on this, the proposed method could allow us to understand neuronal communication through spiking activities at the extracellular recording level.

The effectiveness of the method proposed in this study was ultimately confirmed using spikes detected from neural data obtained from M1 of the rhesus macaque. Given the absence of a definite ground truth in actual neural data, our focus was on assessing whether the ISI distribution aligns with the shape and its magnitude of the gamma distribution. Overlapping spikes were detected and decomposed in a similar manner as in the simulation, then included in sorted spike clusters for ISI analysis. Our findings indicate that the ISI of spikes obtained through the proposed method not only more discovers valid spikes but also well conforms to the gamma function along with those obtained with LG.

Through this study, we investigated the potential of identifying overlapping neural spikes with a synthetic data-driven classifier and leveraging them via a heuristic optimization algorithm. This presents an innovative opportunity to develop and assess models under conditions where ground truth is challenging to ascertain, such as in neural data. It will open avenues for uncovering hidden information that may otherwise go unnoticed, particularly in scenarios involving tactile stimulation and rapid eye movement changes. Furthermore, we introduced a method to compute spike templates by robustly estimating isolated spike clusters through a combination of subspace learning-based feature distribution and iForest-based waveform sample analysis. This approach will be applicable not only to the method proposed in this study but also to template matching-based spike sorting technologies in noisy environments.

Reflecting on our study, we have demonstrated a significant advancement in neural data analysis by addressing the challenge of identifying overlapping spikes without definitive ground truth. Our employment of unsupervised domain adaptation and synthetic spikes has refined neural spike classification, contributing to the broader field of neural engineering. Looking forward, our findings lay the groundwork for further exploration of diverse neural signal types, potentially enriching our understanding of neural dynamics and aiding in the development of more sophisticated models.

## Supplementary Information

Supplementary Material 1.

## Data Availability

All data generated or analyzed during this study are included in this article.

## Abbreviations

tSNE: T-distributed stochastic neighbor embedding
PCA: Principal component analysis
LDA: Linear discriminant analysis
GMM: Gaussian mixture model
iForest: Isolation forest algorithm
ISI: Interspike interval
PSO: Particle swarm optimization
SNR: Signal-to-noise ratio
M1: Primary motor cortex
iTrees: Isolation trees
SVM: Support vector machine
RBF: Radial basis function
RMSE: Root-mean square error

## References

[1] Rey HG, Pedreira C, Quian Quiroga R. Past, present and future of spike sorting techniques. Brain Res Bull. 2015;119:106–17. 25931392 10.1016/j.brainresbull.2015.04.007 PMC4674014

[2] Hawkes AG. Spectra of some self-exciting and mutually exciting point processes. Biometrika. 1971;58:83–90.

[3] Nádasdy Z, Hirase H, Czurkó A, Csicsvari J, Buzsáki G. Replay and time compression of recurring spike sequences in the hippocampus. J Neurosci. 1999;19:9497–507. 10531452 10.1523/JNEUROSCI.19-21-09497.1999 PMC6782894

[4] Huang L, Gan L, Ling BW-K. A unified optimization model of feature extraction and clustering for spike sorting. IEEE Trans Neural Syst Rehabil Eng. 2021;29:750–9. 33877983 10.1109/TNSRE.2021.3074162

[5] Singer W. Synchronization of cortical activity and its putative role in information processing and learning. Annu Rev Physiol. 1993;55:349–74. 8466179 10.1146/annurev.ph.55.030193.002025

[6] Nocon JC, Gritton HJ, James NM, Mount RA, Qu Z, Han X, Sen K. Parvalbumin neurons enhance temporal coding and reduce cortical noise in complex auditory scenes. Commun Biol. 2023;6:751. 37468561 10.1038/s42003-023-05126-0 PMC10356822

[7] Tiesinga P, Fellous J-M, Sejnowski TJ. Regulation of spike timing in visual cortical circuits. Nat Rev Neurosci. 2008;9:97–107. 18200026 10.1038/nrn2315 PMC2868969

[8] Xiang Z, Huguenard JR, Prince DA. Cholinergic switching within neocortical inhibitory networks. Science. 1998;537:985–8. 10.1126/science.281.5379.985 9703513

[9] Jang HJ, et al. Distinct roles of parvalbumin and somatostatin interneurons in gating the synchronization of spike times in the neocortex. Sci Adv. 2020;6: eaay5333. 32426459 10.1126/sciadv.aay5333 PMC7176419

[10] Sakurai Y, Takahashi S. Dynamic synchrony of firing in the monkey prefrontal cortex during working-memory tasks. J Neurosci. 2006;26:10141–53. 17021170 10.1523/JNEUROSCI.2423-06.2006 PMC6674631

[11] Luo J, et al. Neural timing of stimulus events with microsecond precision. PLoS Biol. 2018;16: e2006422. 30365484 10.1371/journal.pbio.2006422 PMC6221347

[12] Chiarion G, Mesin L. Resolution of spike overlapping by biogeography-based optimization. Electronics. 2021;10:1469.

[13] Mokri Y, et al. Sorting overlapping spike waveforms from electrode and tetrode recordings. Front Neuroinform. 2017;11:53. 28860985 10.3389/fninf.2017.00053 PMC5562672

[14] Yeganegi H, Salami P, Daliri MR. A template-based sequential algorithm for online clustering of spikes in extracellular recordings. Cogn Comput. 2020;12:542–52.

[15] Todorova S, et al. To sort or not to sort: the impact of spike-sorting on neural decoding performance. J Neural Eng. 2014;11: 056005. 25082508 10.1088/1741-2560/11/5/056005 PMC4454741

[16] Won DS, Chong DY, Wolf PD. Effects of spike sorting error on information content in multi-neuron recordings. In: 1st international IEEE EMBS conference on neural engineering 2003. Capri Island: IEEE; 2003. p. 618–21.

[17] Shao P-C, et al. Effects of spike sorting error on the Granger causality index. Neural Netw. 2013;46:249–59. 23845518 10.1016/j.neunet.2013.06.001

[18] Wouters J, Kloosterman F, Bertrand A. A data-driven spike sorting feature map for resolving spike overlap in the feature space. J Neural Eng. 2021;18:0460a7. 10.1088/1741-2552/ac0f4a 34181592

[19] Abeles M, Goldstein MH. Multispike train analysis. Proc IEEE. 1977;65:762–73.

[20] Lewicki MS. A review of methods for spike sorting: the detection and classification of neural action potentials. Network. 1998;9:R53–78. 10221571

[21] Mahallati S, et al. Cluster tendency assessment in neuronal spike data. PLoS ONE. 2019;14: e0224547. 31714913 10.1371/journal.pone.0224547 PMC6850537

[22] Keshtkaran MR, Yang Z. Noise-robust unsupervised spike sorting based on discriminative subspace learning with outlier handling. J Neural Eng. 2017;14: 036003. 28198354 10.1088/1741-2552/aa6089

[23] Liu M, et al. Classification of overlapping spikes using convolutional neural networks and long short term memory. Comput Biol Med. 2022;148: 105888. 35872414 10.1016/j.compbiomed.2022.105888

[24] Liu FT, Ting KM, Zhou Z-H. Isolation forest. In: Eighth IEEE international conference on data mining (ICDM). Pisa: IEEE; 2008. p. 413–22.

[25] Kennedy J, Eberhart R. Particle swarm optimization. In: International conference on neural networks, vol. 4. Perth: IEEE; 1995. p. 1942–8.

[26] Bagshaw EV, Evans MH. Measurement of current spread from microelectrodes when stimulating within the nervous system. Exp Brain Res. 1976;25:391–400. 182517 10.1007/BF00241729

[27] Halliday D, Resnick R. Physics for students of science and engineering (combined edition). New York: Wiley; 1962.

[28] Shinomoto S, Miura K, Koyama S. A measure of local variation of inter-spike intervals. Biosystems. 2005;79:67–72. 15649590 10.1016/j.biosystems.2004.09.023

[29] Shimokawa T, Koyama S, Shinomoto S. A characterization of the time-rescaled gamma process as a model for spike trains. J Comput Neurosci. 2010;29:183–91. 19844786 10.1007/s10827-009-0194-y

[30] Kim KH, Kim SJ. Neural spike sorting under nearly 0-dB signal-to-noise ratio using nonlinear energy operator and artificial neural-network classifier. IEEE Trans Biomed Eng. 2000;47:1406–11. 11059176 10.1109/10.871415

[31] Quiroga RQ, Nadasdy Z, Ben-Shaul Y. Unsupervised spike detection and sorting with wavelets and superparamagnetic clustering. Neural Comput. 2004;16:1661–87. 15228749 10.1162/089976604774201631

[32] Bishop C. Pattern recognition and machine learning. Cham: Springer; 2006.

[33] Kreuz T, Bozanic N, Mulansky M. SPIKE-Synchronization: a parameter-free and time-resolved coincidence detector with an intuitive multivariate extension. BMC Neurosci. 2015;16:P170.

[34] Quian Quiroga R, Kreuz T, Grassberger P. Event synchronization: a simple and fast method to measure synchronicity and time delay patterns. Phys Rev E. 2002;66: 041904. 10.1103/PhysRevE.66.041904 12443232

