# Supplementary material for: Synthetic data-driven overlapped neural spikes sorting: decomposing hidden spikes from overlapping spikes
Source: Mol Brain. 2024 Nov 28;17:89. doi: 10.1186/s13041-024-01161-y (PMC11606139; doi:10.1186/s13041-024-01161-y)
Supplement: Supplementary file 1 — Supplementary Material 1. [file 13041_2024_1161_MOESM1_ESM.pdf]

Supplementary Materials:

Synthetic Data-driven Overlapped Neural Spikes Sorting: Decomposing Hidden Spikes from Overlapping Spikes

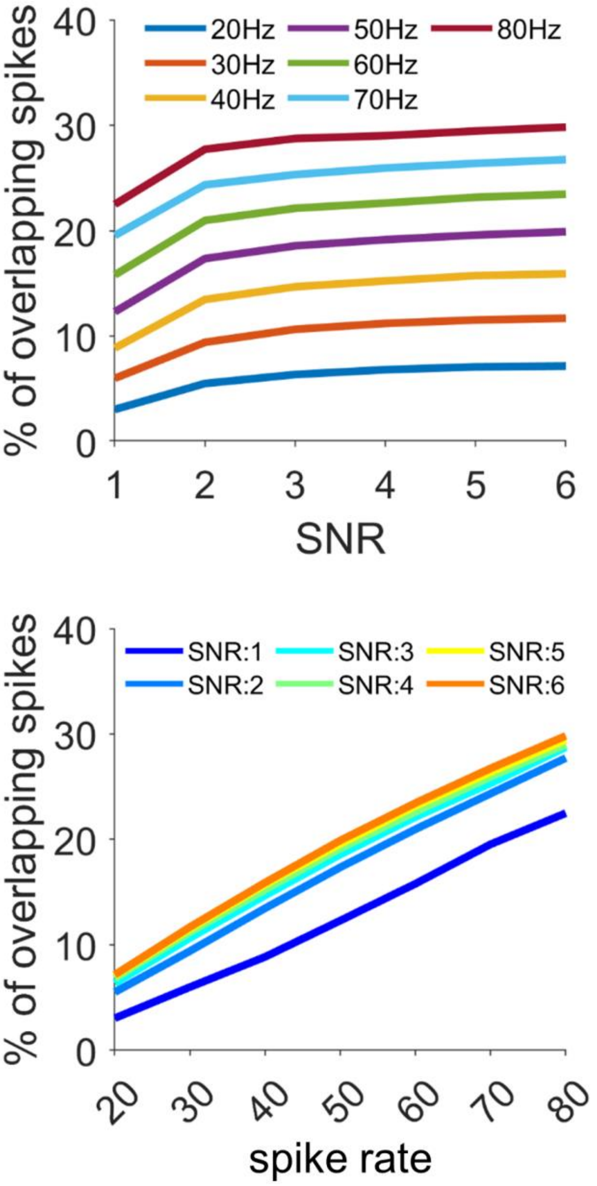

Figure S1. Percentage of overlapping spikes depending on changes in the signal-to-noise ratio (when spike rates are uniformly distributed). The top panel illustrates the change in the proportion of overlapping spikes as the SNR varies during synthetic spike generation. Each colored line corresponds to the initially set spike rates. The bottom panel depicts the change in the proportion of overlapping spikes as the spike rate varies. Each colored line represents the SNR condition during synthetic spike generation.

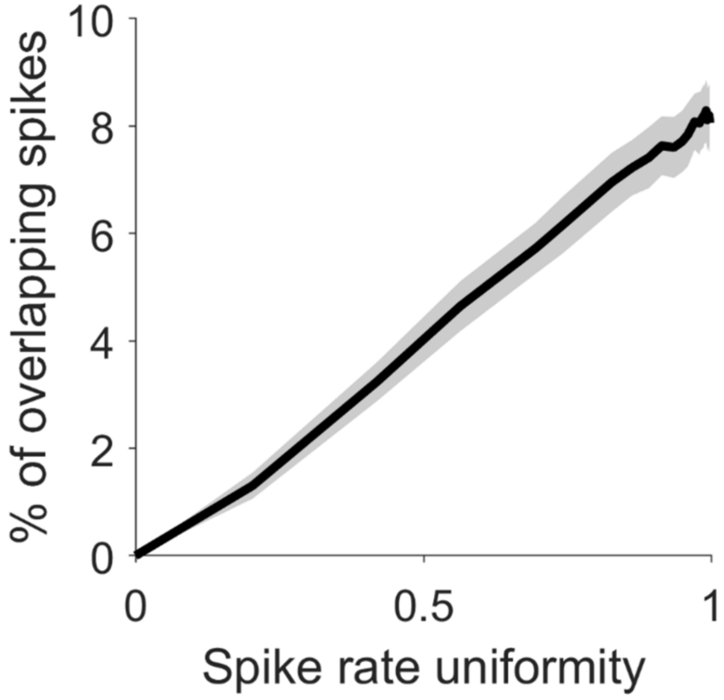

Figure S2. A relationship between the percentage of overlapping spikes and uniformities of spike rate for each single unit. Solid dark line and shaded patch denote the mean and the standard error across the repeated simulations, respectively.

In this simulation, uniformity can be calculated using the normalized entropy of the spike rates for each single unit. The spike rate uniformity can thus be expressed as:

$$\mathbf{Uniformity} = -\frac{\sum_{j=1}^{J} \boldsymbol{p_j} \log_2 \boldsymbol{p_j}}{\log_2 \boldsymbol{J}}$$

where p_j represents the probability mass function of the spike rates, z_j, per second for J units. To generate spike trains constrained to each level of uniformity, we initialized each unit's spike rate by linearly transforming the spike rates of uniformly distributed units, increasing the slope, h, from 0 to 1 at intervals of 0.1 across the unit sequence. The maximum spike rates of all units were fixed at 30 Hz, and the SNR was set to 2. The transformed spike rate for each unit can be expressed as:

$$\hat{\boldsymbol{z}}_j = \boldsymbol{h z_j}$$

where $\hat{\boldsymbol{z}}_j$ represents the transformed spike rate for unit j, which can be adjusted between 0 and 30 Hz for each unit, leaving all other units unchanged. These simulations for each uniformity condition were repeated 500 times.

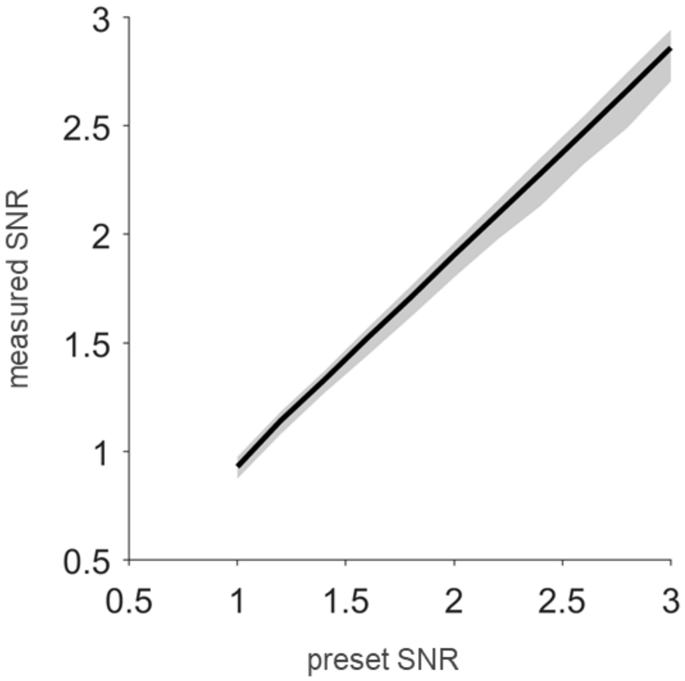

Figure S3. A relationship between the preset SNR and the measured SNR of synthetic spikes. Solid dark line and shaded patch denote the mean and the standard error across the repeated simulations, respectively.

The difference between the preset SNR and the measured SNR is due to two main factors: the randomly sampled distances between neurons and electrodes, set to match the number of spike templates, and the randomly sampled scale parameter of the gamma distribution. The correlation coefficient between them is 0.99 ($p < 0.01$), indicating a very strong correlation.
